# Metabolic alterations underlying Bevacizumab therapy in glioblastoma cells

**DOI:** 10.18632/oncotarget.21761

**Published:** 2017-10-10

**Authors:** Vera Miranda-Gonçalves, Diana Cardoso-Carneiro, Inês Valbom, Fernanda Paula Cury, Viviane Aline Silva, Sara Granja, Rui M. Reis, Fátima Baltazar, Olga Martinho

**Affiliations:** ^1^ Life and Health Sciences Research Institute (ICVS), School of Medicine, University of Minho, Campus de Gualtar, Braga, Portugal; ^2^ ICVS/3Bs-PT Government Associate Laboratory, Braga/Guimarães, Portugal; ^3^ Molecular Oncology Research Center, Barretos Cancer Hospital, Barretos, São Paulo, Brazil

**Keywords:** anti-angiogenic therapy, Bevacizumab, glioblastoma, glycolytic metabolism

## Abstract

Anti-VEGF therapy with Bevacizumab is approved for glioblastoma treatment, however, it is known that tumors acquired resistance and eventually became even more aggressive and infiltrative after treatment. In the present study we aimed to unravel the potential cellular mechanisms of resistance to Bevacizumab in glioblastoma *in vitro* models.

Using a panel of glioblastoma cell lines we found that Bevacizumab is able to block the secreted VEGF by the tumor cells and be internalized to the cytoplasm, inducing cytotoxicity *in vitro*. We further found that Bevacizumab increases the expression of hypoxic (HIF-1α and CAIX) and glycolytic markers (GLUT1 and MCT1), leading to higher glucose uptake and lactate production. Furthermore, we showed that part of the consumed glucose by the tumor cells can be stored as glycogen, hampering cell dead following Bevacizumab treatment. Importantly, we found that this change on the glycolytic metabolism occurs independently of hypoxia and before mitochondrial impairment or autophagy induction. Finally, the combination of Bevacizumab with glucose uptake inhibitors decreased *in vivo* tumor growth and angiogenesis and shift the expression of glycolytic proteins.

In conclusion, we reported that Bevacizumab is able to increase the glucose metabolism on cancer cells by abrogating autocrine VEGF *in vitro*. Define the effects of anti-angiogenic drugs at the cellular level can allow us to discover ways to revert acquired resistance to this therapeutic approaches in the future.

## INTRODUCTION

Gliomas are the most common group of brain tumors, representing approximately 70%. Glioblastoma (GBM) is the most common and aggressive glioma in adults [1–4]. Standard treatment consists of surgical resection followed by radiotherapy plus concomitant and adjuvant temozolomide chemotherapy. However, this regimen offers modest benefits with a median overall survival of only 16 months [4–8]. Therefore, a better understanding of GBM biology and more effective therapeutic options are warranted. In the recent years, new strategies to improve cancer treatment, such as the usage of molecular targeted therapies, that selectively target altered proteins in cancer cells, have been developed [9].

GBM is one of the most vascularized human tumors, with disorganized tumor vessels, highly permeable and presenting abnormal endothelial walls [10, 11]. Therefore, they have a high angiogenic capacity producing a variety of pro-angiogenic factors, including VEGF (Vascular endothelial growth factor) [12, 13]. In the last years, VEGF-A became an attractive strategy for targeting angiogenesis in cancer patients [1], since is the most common isoform present in tumor tissues, being also associated with patient’s worse prognosis [13].

Bevacizumab (Avastin® - Beva), a humanized anti-VEGF monoclonal antibody, was the first approved anti-VEGF therapy by the United States Food and Drug Administration (FDA) in 2004. It was first approved to metastatic colorectal cancer and non-small cell lung cancer treatment in combination with other chemotherapeutic agents [14, 15]. In GBM, Beva was approved as a second line treatment option in combination with chemotherapy [16, 17]. Currently, is in phase III clinical trials as first line usage in GBM treatment. In the phase II trials, alone or in combination with standard therapy, it was observed that Beva clearly increases the radiographic response on magnetic resonance imaging, resulting in an increased progression free survival of 6 months. Conversely, the impact on overall survival was unclear [18–23]. Thus, the clinical benefits of anti-VEGF therapy in most patients are transient and followed by a restoration of angiogenesis, tumor progression and invasion [24].

Recently, several reports have demonstrated that the metabolic rewiring of cancer cells can also be responsible for resistance to oncogene-targeted therapies (reviewed in [25]). Accumulating evidences have also associated the reprogramming of metabolism in the acquired resistance to Beva treatment [26, 27]. The *in vivo* studies have shown that the reduced perfusion, associated with Beva treatment, causes a decrease on nutrients and oxygen supply, leading to the development of hypoxia in the tumor microenvironment [24, 26–28], and consequently to the remodeling of glycolytic metabolism [26–37].

The cellular mechanisms through which metabolic alterations occur on tumor cells upon VEGF-targeted therapies are still unknown. Accordingly, the major aim of this work was to study whether these metabolic alterations can occur already at the cellular level and independently of tumor microenvironment or hypoxia, trying to propose new tools, such as *in vitro* models, to study ways overcome Beva associated resistance.

## RESULTS

### Bevacizumab is internalized by GBM cells and impairs *in vitro* cell viability

**Figure 1:**
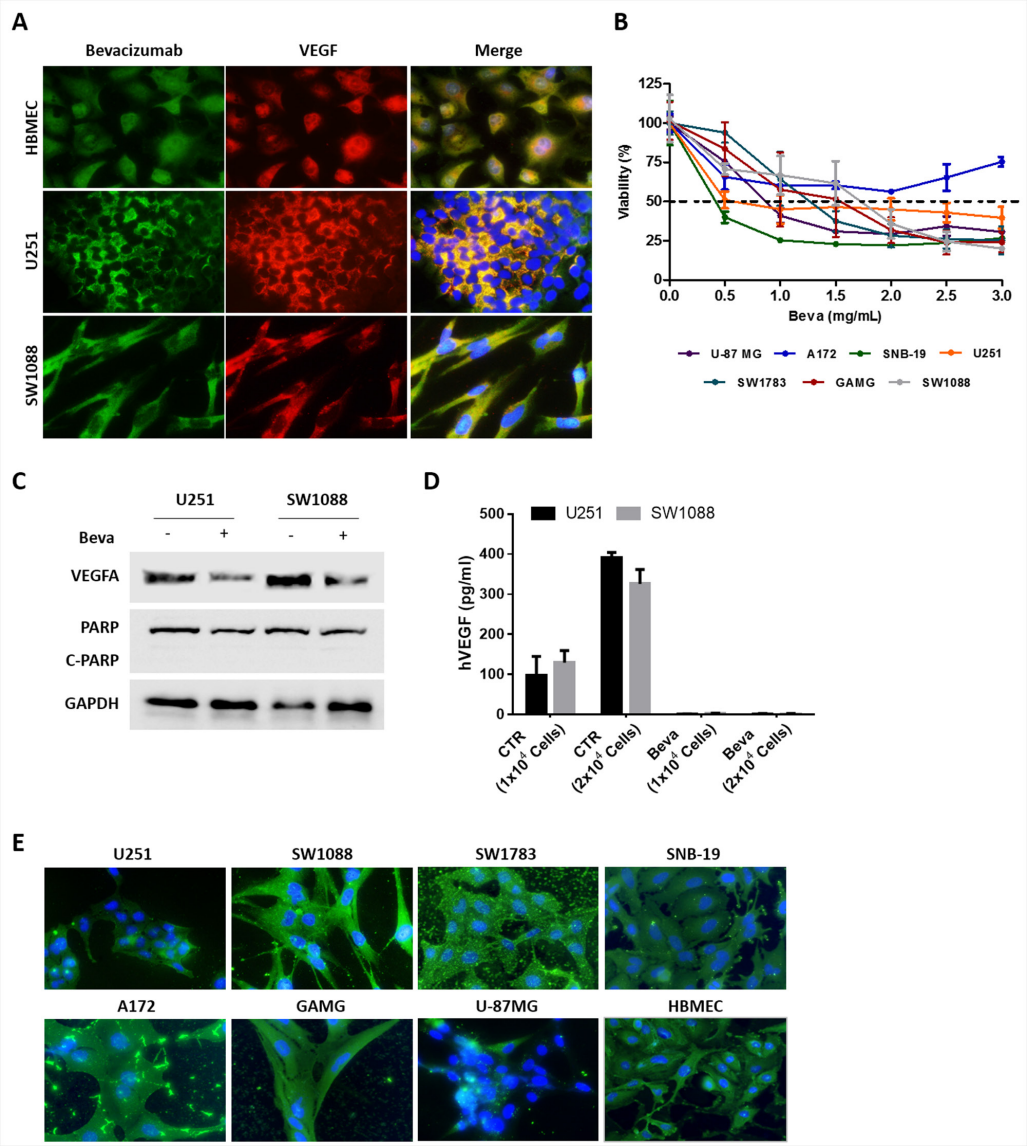
*In vitro* effect of Bevacizumab in GBM cell lines **(A)** Immunofluorescence for VEGFA using a specific anti-VEGF antibody and Beva as primary antibodies. **(B)** Cell viability of GBM cell lines exposed to increasing concentrations of Beva was assessed by MTS assay at 72 hours of treatment; results are from three independent assays, each one in triplicates. **(C)** Western Blot analysis for VEGF and the apoptotic marker (PARP cleavage). The cells were treated with 2 mg/ml of Beva during 24 hours. **(D)** ELISA assay for human VEGF. The cells were seeded in different numbers and the supernatant of the cells was collected after 24 hours of Beva treatment (2 mg/ml) for VEGF quantification. **(E)** Beva internalization was evaluated in GBM cell lines treated during 24 hours with Beva (2 mg/ml) by using an anti-human IgG antibody. On the images from (A) and (E) the cell nucleus were counterstained with DAPI and the pictures were taken at 400x in an Olympus fluorescence microscope.

Beva is a monoclonal antibody designed to target specifically the ligand VEGF-A. This ligand, in the tumor microenvironment, is supposedly secreted to the extracellular region to stimulate VEGF receptors that are present in endothelial cells. Thus, in the present work we initially aimed to determine whether GBM tumor cells secret VEGF or whether they can be expressing VEGF intracellularly, and how Beva can enter the cell and recognize it *in vitro*. For that we co-stained two GBM cell lines, and one brain endothelial cell line (HBMEC) as control, with VEGF and Beva as primary antibodies. We found that all cell lines express VEGF in the cytosol and that Beva recognizes an intracellular protein that co-localizes exactly with VEGF, as expected (Figure 1A).

In order to determine the cytotoxic effect of Beva, a panel of GBM cell lines were treated with increasing concentrations of Beva during 72 hours and cellular viability was assessed by MTS assay. Excepting A172 cells, all the other cell lines reached an IC_50_ value of up to 1mg/ml (Figure 1B).

To further confirm its functionality *in vitro*, the cells were treated by 24 hours with a non-cytotoxic dose of Beva, as showed by the absence of PARP cleavage (Figure 1C). We found that the intracellular VEGF was decreased after Beva treatment, as shown by a diminishment of the protein levels by western blot (Figure 1C). In addition, by ELISA assay we showed that GBM cells secret VEGF proportionally to the number of existing cells, being that ligand completely abolished from the culture medium after Beva treatment (Figure 1D).

Next, we wondered whether Beva could be internalized by the tumor cells. For that we treated all cell lines with Beva and used an anti-human IgG antibody to follow its location. As it can be observed on Figure 1E, we found that Beva is internalized, being located mainly at the cytoplasm, presenting in some cell lines a dot-like staining (Figure 1E). Moreover, it can be observed in some cell lines (e.g. GAMG, SW1783 and A172) an accumulation of Beva at the extracellular region (attached to the coverslip).

Overall, these findings re-enforces our hypothesis that Beva can be used *in vitro*, being functional and having biological and cytotoxic activity. Importantly, this *in vitro* effect of Beva was not specific for GBM, occurring also in lung and colorectal cell lines (Supplementary Figure 1).

### Bevacizumab treatment alters the glycolytic metabolism of glioblastoma cells

To determine whether Beva promotes the glycolytic metabolism independently on oxygen depletion, and consequently hypoxia, we used GBM *in vitro* models by selecting two cell lines with different metabolic behaviors; U251 as one of the most glycolytic GBM cell line, and SW1088, as a more oxidative cell line, as previously described by our group [38, 39].

**Figure 2:**
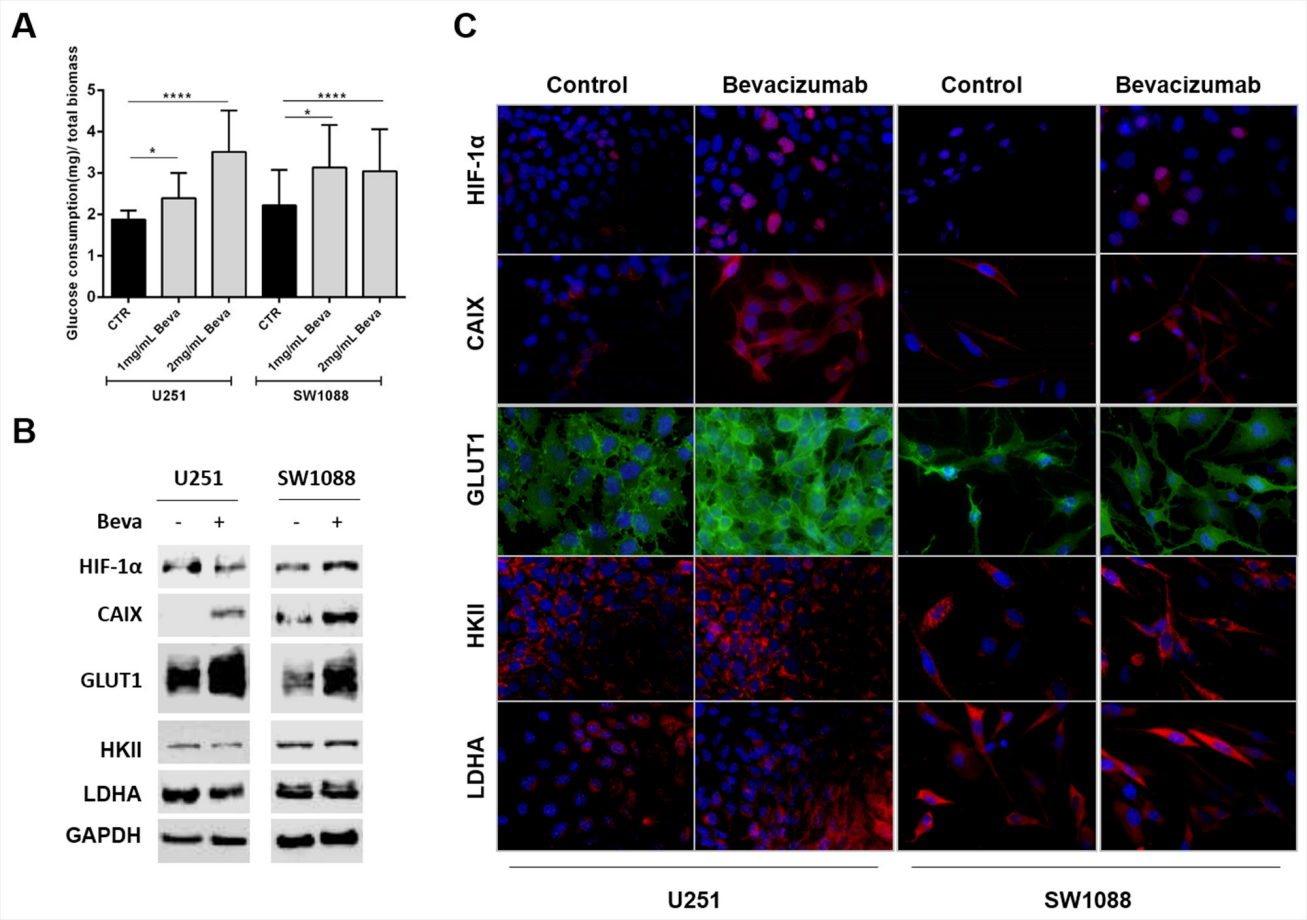
Effect of Bevacizumab treatment on glucose metabolism of GBM cells **(A)** Beva increased the glucose consumption of U251 and SW1088 cells after 48 hours of treatment; results are representative of three independent experiments, each one in triplicates; *p<0.05, ****p≤0.0001 Beva vs control. Western blot **(B)** and immunofluorescence **(C)** of hypoxia markers and glycolytic markers in U251 and SW1088 cells treated with 2mg/ml Beva during 48 hours. Increased HIF-1α, CAIX and GLUT1 expression was observed after treatment. The cell nucleus were counterstained with DAPI and the pictures were taken at 400x in an Olympus fluorescence microscope.

Beva treatment increased significantly the glucose consumption in the two cell lines (Figure 2A), an increase that was accomplished by an increased expression of hypoxic markers, such as HIF-1α (hypoxia-inducible factor 1-alpha) and CAIX (Carbonic anhydrase 9) (Figure 2B and 2C), as well as an increase in the glycolytic markers (Figure 2B), being mainly verified a preferential location of GLUT1 (glucose transporter 1) at the plasma membrane (Figure 2C). No alterations on the expression of other metabolic markers, such as hexokinase II (HKII) and lactate dehydrogenase (LDHA), were observed both by western blot or immunofluorescence (Figure 2B and 2C).

**Figure 3:**
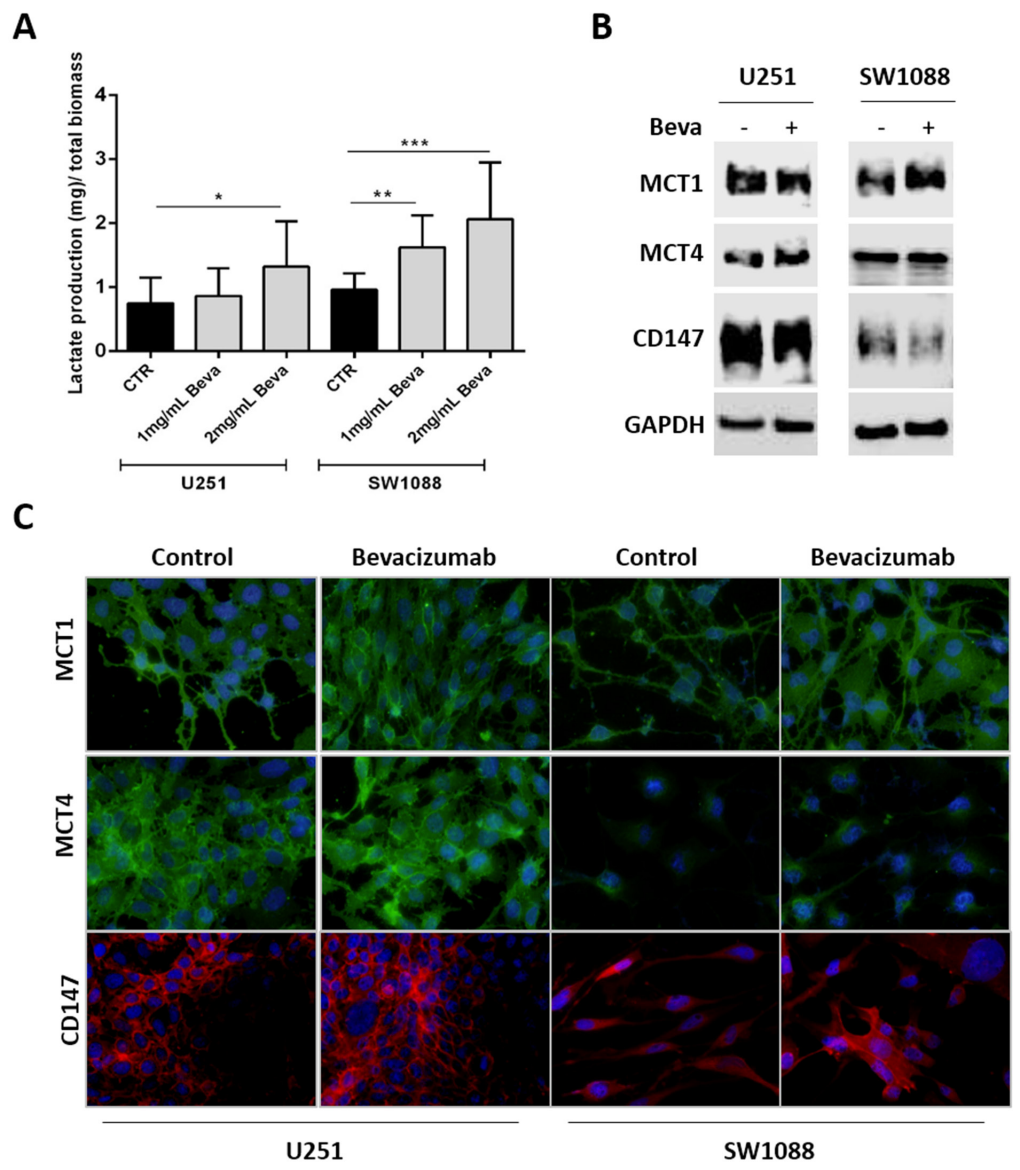
Modulation of lactate production and transport in Bevacizumab treated GBM cells **(A)** The lactate export is increased in U251 and SW1088 cells treated with 2mg/ml of Beva by 48 hours; results are representative of three independent experiments, each one in triplicates; *p<0.05, **p<0.01, ***p≤0.001 Beva *vs* control. MCT1expression, but not MCT4 and CD147, increased in SW1088 treated with 2mg/ml Beva during 48 hours by Western blot **(B)** and immunofluorescence (the cell nucleus were counterstained with DAPI and the pictures were taken at 400x in an Olympus fluorescence microscope) **(C)**.

Furthermore, since it was observed an increase on glucose uptake and glycolytic markers expression upon Beva treatment, we intended to study whether this drug can promote Warburg effect by increasing the levels of lactate production (Figure 3). Thus, we observed that only 2mg/ml Beva increased the lactate production on U251 cells, whereas both concentrations of Beva (1mg/ml and 2mg/ml) were able to significantly increase the lactate production on SW1088 cells (Figure 3A). Additionally, an increase on the lactate transporter MCT1 (monocarboxylate transporters 1) at the plasma membrane was observed in both cell lines (Figure 3B and 3C). Neither the transporter MCT4 (monocarboxylate transporters 4) nor its chaperone CD147 were altered upon Beva treatment in both cell lines (Figure 3B and 3C).

The effect of Beva on glucose metabolism was also confirmed in colon and lung cancer cell lines (Supplementary Figure 2).

### Bevacizumab treatment promotes glycogen accumulation as a potential survival mechanism

**Figure 4:**
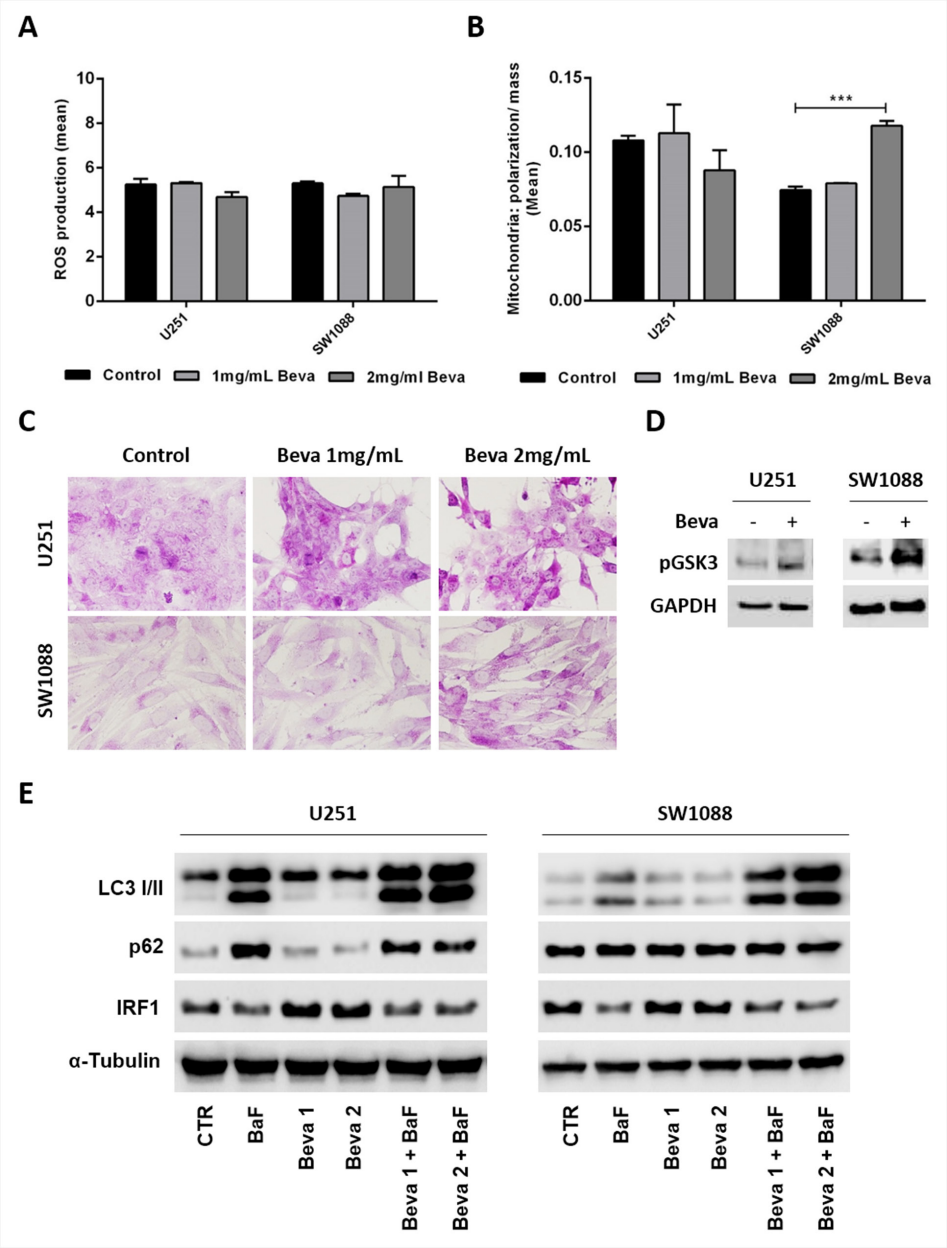
Pro-survival mechanism activated by Bevacizumab in GBM cells Cells treatment with 2mg/ml of Beva during 48 hours did not diminished the ROS production **(A)** and the ratio mitochondrial polarization/mitochondrial mass **(B)**, the results are representative of three independent experiments. **(C)** Treatment of cells with 2 mg/ml of Beva increased the glycogen accumulation after 48 hours; representative pictures of PAS staining were taken at 400x in an Olympus BX16 microscope; **(D)** Expression of phosphorylated Glycogen synthase kinase 3 (GSK3) also increased after Beva 2mg/ml treatment during 48 hours by western blot. **(E)** Western blot was also done for the autophagic markers LC3 I/II, p62 and IRF1 in U251 and SW1088 cells treated with 1 mg/ml and 2mg/ml of Beva during 48 hours. Bafilomycin (BaF) was used to induce autophagy. The results showed that Beva has no impact on autophagy induction nor inhibition in the two cell lines.

To understand whether the changes on the glycolytic metabolism driven by Beva can be due mitochondria impairment, we assessed mitochondrial activity and production of reactive oxygen species (ROS). We observed that Beva did not induce significant changes in ROS production (Figure 4A), nor in the ratio of mitochondrial polarization/mitochondrial mass in U251 cell line, but increases mitochondrial activity in SW1088 cells after 48 hours of Beva treatment at 2 mg/ml (Figure 4B).

It is well described that cells undergoing hypoxia have the ability to store the consumed glucose into glycogen as a survival mechanism [40]. It is also known that hypoxia-mediated autophagy promotes tumor cell survival [41]. Since Beva does not show a significant impact on mitochondrial impairment, and we have observed that the glucose consumed was not all converted into lactate (comparing graphs from Figure 2 and 3), we screened for alterations on glycogen synthesis and autophagy induction after Beva treatment. By PAS staining we observed an increase on glycogen accumulation on cells treated during 48 hours with Beva, an effect that seems to be dose dependent (Figure 4C). Accordingly, we verified an increase on glycogen synthase (GSK3) activation by increased expression of its phosphorylated form in both cell lines (Figure 4D). Concerning autophagy, we evaluated LC3I/II conversion and p62 expression by western blot and verified that Beva has no effect in autophagy induction or inhibition in both cell lines (Figure 4E). Moreover, a recent *in vitro* experiment showed that Beva treatment increased IRF1 (Interferon-regulatory factor-1) expression in a dose and time dependent manner, which was coincident with Beva-mediated autophagy [42]. Thus, to confirm the lack of effect of Beva in autophagy induction we assessed also the levels of IRF1 by western blot (Figure 4E). Curiously, IRF1 levels seems to be unaltered in SW1088 cell line, and moderately increased in U251 upon Beva treatment, but is significantly decreased in both cell lines when autophagy is induced (by bafilomycin treatment) (Figure 4E).

### *In vivo* tumor growth inhibition is pronounced when Bevacizumab is combined with glucose uptake blockers

**Figure 5:**
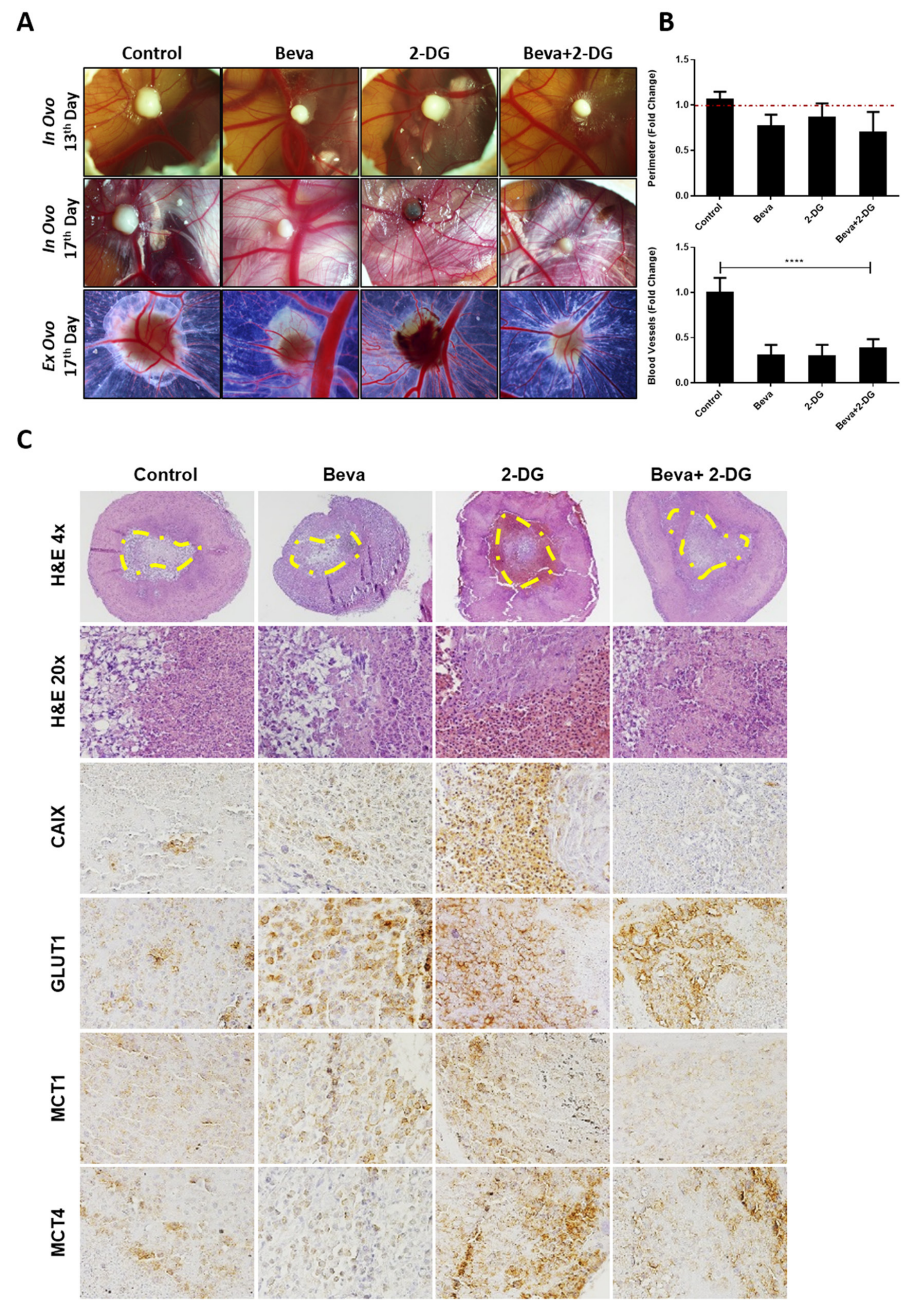
*In vivo* effect of Bevacizumab in combination with glucose analogue 2-DG in U251 induced tumors **(A)** *In ovo* pictures at 13^th^ day of embryo development (4 days of tumor growth) and at 17^th^ day (4 days of treatment, *in ovo* and *ex ovo*); representative pictures were taken at 20x in a Zeiss stereomicroscope. **(B)** Graphical representation of tumors perimeter (in relation to day 13 that was considered as 1 in each group) at the upper panel and of blood vessels (in relation to control without drugs, considered as 1) in the lower panel, showing a slight decrease on tumor size and significantly decrease in blood vessels on tumors treated with both Beva (2 mg/ml), 2-DG (1 mM) alone and 2-DG plus Beva. The results are representative for a media of 8 eggs per group; ****p<0.001 treated group *vs* control for blood vessels counting. **(C)** H&E and IHC expression of hypoxia and metabolic markers after 4 days of treatment with Beva, 2-DG and 2-DG+Beva. It is observed in the H&E a higher number of necrotic cells in the tumors that were treated with 2-DG and with Beva+2-DG. The IHC shows a decrease in CAIX expression in the combination group; GLUT1 increased in all groups; MCT1 expression increased after Beva treatment and decreased in combination group; MCT4 expression increased only in 2-DG conditions. The H&E pictures were taken with 4 and 20X objective, while and the ones from IHC were taken with 200X amplification in an Olympus microscope. The yellow line defines the necrotic area of U251 micro-tumors.

Our findings suggested that *in vitro* Beva treatment induces metabolic alterations by increasing glucose consumption, lactate production and glycogen synthesis. Accordingly, we further aimed to study whether combination of Beva with a metabolic inhibitor can potentiate its effect and revert the metabolic status of the tumors. For that we performed the *in vivo* CAM assay, by using U251 cell line, and treated the tumors with Beva and 2-DG (glucose analogue) alone, or both in combination. Overall, there is a slight decrease in the tumor mass in all the treatment conditions, mainly when Beva was combined with 2-DG (Figure 5A and 5B), although not reaching statistical significance. Concerning the effect of the agents on angiogenesis, we verified that all of them were extremely effective in reducing the tumors recruitment of blood vessels (Figure 5A and 5B).

The histological analysis of the CAM-formed tumors showed a central necrotic region in all experimental conditions (Figure 5C – HE 4x). Accordingly, we were able to confirm a higher number of death/necrotic cells in the tumors that were treated with 2-DG and with Beva+2-DG (Figure 5C – HE 20x). By immunohistochemistry, we observed that the expression of hypoxic marker CAIX increased with either Beva or 2-DG treatment alone, and is weaker in the combination of Beva+2-DG (Figure 5C). We also observed an increase on GLUT1 expression after Beva treatment, as well as with 2-DG, with no changes in the combination group (Figure 5C). Regarding MCT expression, there was an increase in MCT1 expression, but not in MCT4, after Beva treatment alone. Combination of Beva + 2-DG decreased the MCT1 expression, however increased the MCT4 expression, probably due to the presence of 2-DG, condition in what we have also observed high levels of MCT4 (Figure 5C).

## DISCUSSION

In the present study, aiming to unravel potential cellular mechanisms of resistance to anti-angiogenic drugs, we wondered whether Beva is able to induce glycolytic alterations *in vitro* and independently of oxygen deprivation. First, we observed that GBM cell lines express and secret VEGF, as previously described [43], and found that Beva is able to sequestrate all the VEGF secreted, decreasing in this way the cellular viability in a dose dependent manner. Importantly, we showed for the first time that Beva can be internalized by different cell lines. Ideally, antibodies suited to therapeutic applications must bind to and actively internalize their cognate target [44, 45]. Thus, we demonstrated here that Beva is functional *in vitro* and have a specific cytotoxic effect by totally abrogating the autocrine function of VEGF on tumor cells, corroborating the pertinence of its study *in vitro*.

By using two GBM cell lines with different metabolic behaviors [38, 39], we prove that Beva treatment promotes metabolic alterations on tumor cells, which are totally independent of hypoxia induction. Specifically, we found that Beva increases the glucose uptake and GLUT1 expression as well as an increase in lactate production. These observations corroborate the *in vivo* mouse studies, where Beva leads to anaerobic metabolism increasing the levels of lactate, alanine, choline and some lipids, as well as to the increase of enzymes associated to glycolytic pathway, like GLUT3, HKII, LDHA and also enzymes associated to pentose phosphate pathway [29, 37].

In addition, beyond increased expression of hypoxic markers (HIF-1α and CAIX), that were already described in *in vivo* models [29, 30, 33, 37], we observed modulation of MCTs expression after Beva treatment, mainly the preferential location of MCT1 at the plasma membrane. Few studies reported modulation of MCT isoforms expression during anti-angiogenic therapy. A study of Curtarello M. *et al.* in ovary cancer, showed that Beva treatment increase MCT4 expression in most oxidative cells, without effect on glycolytic ones, and increased MCT1 expression, both in tumor and liver metastasis, only after Beva long term treatment [36]. In our study, the preferential increase of MCT1 upon Beva treatment could be explained by the knowledge that GBM cells prefer to use MCT1 for lactate efflux, in contrast to other tumors that prefer MCT4 [38, 39].

In addition to alterations on glucose metabolism, other metabolic alterations are known to occur in Beva treated tumors, which are concomitant with the upregulation of HIF-1α. In colorectal *in vivo* models, the metabolic assays revealed a significant impairment of mitochondrial function upon Beva treatment [26], and a significant increase in cellular senescence associated with upregulation of p16 *in vitro* [46]. In contrast, herein we found that Beva has no impact on mitochondrial function or mass in U251 GBM cell line, but increase mitochondrial activity in the most oxidative one, the SW1088 cell line. Since our experiments were done in normoxia conditions, and that Beva increases the glucose consumption, is also expectable an increase on mitochondrial function in the more oxidative cell lines.

In GBM *in vivo* models it was described that hypoxia-mediated autophagy promotes tumor cell survival in patients that are resistant to Beva [41]. Importantly, it was recently shown that interferon-regulatory factor-1 (IRF1) may regulate Beva-induced autophagy in GBM cell lines, and thus independently of hypoxia [42]. Herein, our *in vitro* study in normoxia conditions, showed that in fact Beva can somehow increase the expression levels of IRF1, but the association with an enhanced authophagy was not observed. Even we used the same antibody for IRF1, the cell lines used were different impairing comparisons with the previous work [42]. Importantly, our findings indicate that, at least at the cellular levels, alterations on glucose metabolism occur earlier and at lower doses than the impairment of mitochondria and autophagy induction.

A study of Pelletier J. and colleagues showed that glycogen synthesis is enhanced in GBM cells when exposed to hypoxia, resulting in a large increase in glycogen stores, induction of autophagy and senescence [40]. Knowing that, and since we found that Beva was able to increase the expression of HIF-1α independently of oxygen presence, and that the rates of glucose uptake were higher than the ones of lactate export, we hypothesized whether the glucose is being used for glycogen storage in GBM treated cell lines. In fact, we demonstrated that Beva treatment increase glycogen accumulation and activity of glycogen synthase kinase in GBM cell lines, probably as a survival mechanism imposed by pseudo-hypoxia generation. The glycogen storage could be used as intermediate source for biosynthetic pathways and prevent cell death [40].

The discovery of alterations on glycolytic metabolism as a putative mechanism of resistance to anti-angiogenic therapy, offers the opportunity for new and effective combinatory therapeutic approaches. It is known that suppressing HIF-1α, with low-dose topotecan, potentiates the effects of the antiangiogenic drugs in mouse models of neuroblastoma and GBM [47, 48]. In colorectal cancer xenograft models, treatment of Beva-resistant cells with the glycolysis inhibitor 3-BP resulted in smaller tumor volume and longer survival [26], while Beva and DCA together dramatically blocked tumor growth compared to either drug alone [49], suggesting that glycolysis blockage may also potentiate the therapeutic effect of anti-angiogenic treatment [26, 49]. Inhibition of CAIX expression, as well as its activity in GBM, leads to a synergistic decrease on xenograft tumor growth when combined with Beva [31].

Our results showed that Beva drives an earlier increase on glucose uptake and glycogen storage, before the appearance of other metabolic alterations, therefore, we use 2-DG, an analogue of glucose uptake, as an alternative strategy to enhance the Beva effects *in vivo*. By inducing U251 micro-tumors in *in vivo* CAM assay, we verified that the combined treatment leads to a decrease in tumor growth and angiogenesis, but has no additional effects when compared to Beva alone. Histologically, we observed that the tumors treated with both Beva and 2-DG have decreased expression of CAIX, MCT1 and increased MCT4, suggesting somehow a restoring on the glycolytic alterations driven by Beva treatment alone. However, further *in vivo* studies, mainly in mouse models, are warranted to validate and explore that findings.

In conclusion, that was the first *in vitro* study seeking to understand the effects of Beva on cellular metabolism, independently of the tumor microenvironment. The findings re-enforces our hypothesis that Beva can be used *in vitro*, being functional and having biological and cytotoxic activity. Importantly, we found that Beva is able to alter the cellular metabolism of cancer cells by abrogating VEGF autocrine function. Thus, the exploitation of anti-angiogenic drugs effect at the cellular level can open the opportunity to discover new therapeutic strategies to overcome Beva resistance *in vivo*.

## MATERIALS AND METHODS

### Cell culture and cell lines

Seven immortalized GBM cell lines were used: SW1088, SW1783, U-87 MG and A172 were obtained from ATCC (American Type Culture Collection), SNB-19 and GAMG were obtained from DSMZ (German Collection of Microorganisms and Cell Cultures) and U251 was kindly provided by Professor Joseph Costello. Human Brain Microvascular Endothelial Cells (HBMEC) cell line was kindly provided by Professor Kwang Sik Kim, John Hopkins School of Medicine, Baltimore, USA. All the cell lines were maintained in DMEM supplemented with 10% FBS, at 37°C and 5% CO_2_.

Authentication of cell lines was performed in our lab by short tandem repeat (STR) DNA typing according to the International Reference Standard for Authentication of Human Cell Lines using a panel of 8 (D5S818, D13S317, D7S820, D16S539, vWA, TH01, TPOX and CSF1P0) STR loci plus gender determination (AMEL) [50]. The genotyping confirmed the identity of all cell lines.

### Drugs

Bevacizumab (Avastin®; Genentech) was kindly provided by the Pharmacy Department from Hospital de S. João, Porto. The 2-deoxy-D-glucose (2-DG) was purchased from Sigma-Aldrich, and reconstituted at stock concentration of 1M in water. Bafilomycin A1, (BaF) was purchased from Santa Cruz Biotechnology (sc-201550) at a stock concentration of 1 mM in DMSO.

### Antibodies

For immunofluorescence (IF) and Western blot (WB) assays, we used the following antibodies and dilutions: VEGF [(1:100 dilution (IF), 1:500 dilution (WB), RM 9128-S0; NeoMarkers)]; Bevacizumab (1:100 dilution (IF), Genentech); MCT1 [(1:200 dilution, AB3538P; Chemicon International (IF)), (1:500 dilution; H-1, sc-365501; Santa Cruz Biotechnology (WB))]; MCT4 (1:500 dilution; H-90; sc-50329; Santa Cruz Biotechnology); CD147 (1:500 dilution, 1.BB.218, sc-71038; Santa Cruz Biotechnology); HKII (1:4000 dilution, ab104836, Abcam); GLUT-1 (1:500 dilution, ab15309, Abcam); HIF-1α [(1:100 dilution (IF); 1:500 dilution (WB), 610958, BD Biosciences), LDHA (1:1000 dilution, E9, sc-137243, Santa Cruz Biotechnology), CAIX (1:2000 dilution, ab15086, Abcam); LC3A/B (1:1000 dilution, 4108, Cell Signaling Technology); p62 (1:1000 dilution, sc-28359, Santa Cruz Biotechnology); IRF1 (1:1000 dilution, sc-135952, Santa Cruz Biotechnology); α-Tubulin (1:5000 dilution, B-5-1-2, sc-2394, Santa Cruz Biotechnology) and GAPDH (1:1000 dilution, sc-69778, Santa Cruz Biotechnology).

### Cell viability assay

Glioma cells were plated into 96-well plates in triplicates at a density of 20x10^3^ cells/well and allowed to adhere overnight in complete DMEM medium. Then, cells were treated with increased concentrations of Beva in DMEM 0.5% FBS during 72 hours. The viable cells were quantified using the Cell Titer96 Aqueous cell proliferation assay (Promega). The results were expressed as the mean percentage ± SD of viable cells relatively to control condition (considered as 100% viability). The IC_50_ concentration was calculated by nonlinear regression analysis using the GraphPad Prism software, as previously described [51]. The assay was performed in triplicate at least three times.

### Immunofluorescence

For indirect immunofluorescence the cells were seeded on cover slips at density of 20x10^3^ cells/well, overnight. When necessary, the cells were incubated during 24-48h with 2mg/ml Beva in DMEM 0.5% FBS. Then, cells were fixed and permeabilized in methanol during 20min. After blocking with 5% bovine serum albumin (BSA) for 30min, cells were incubated overnight at room temperature with the primary antibodies. Then, 1h of incubation with the secondary antibody anti-Human IgG-Alexa Fluor 488 (1:500 dilution, A11013, Invitrogen) in 5% BSA was performed for Beva; anti-rabbit-Alexa Fluor 568 (1:500 dilution, A11011, Invitrogen) in 5% BSA was performed for VEGF; anti-rabbit-Alexa Fluor 488 (1:500 dilution, A11008, Invitrogen) in 5% BSA was performed for MCT4, MCT1 and GLUT-1 and anti-mouse-Alexa Fluor 594 antibody (1:250 dilution, A11032, Invitrogen) for CD147, HKII, LDHA and CAXI. Additionally, to see the internalization of Beva, the cells exposed to Beva during 24h, we directly incubated with an anti-Human IgG-Alexa Fluor 488 (1:500 dilution, A11013, Invitrogen) after fixation and blocking.

Finally, after washing in PBS, cells were mounted in Vectashield Mounting Media with 4′,6-diamidino-2-phenylindole (DAPI) (Vector Laboratories) and images were obtained with a fluorescence microscope (Olympus IX81), using the Cell P software.

### ELISA

To quantify the levels of VEGF in the culture medium before and after Beva treatment we used the Human VEGF ELISA Kit (KHG011, Novex), as recommended by the manufacture. The cells were cultured at a density of 10x10^3^ and 20 x10^3^ cells/well in a 12-well plate, and after 24 hours’ treatment with Beva (2mg/ml) the culture medium was collected for ELISA analysis. The results were expressed as mean pg/ml ± SD.

### Western blotting

The cells were seeded in 6-well plate at density of 2.5x10^5^ cells/well and allowed to adhere overnight. After, 48hours of 2mg/ml Beva treatment, cells were collected for protein extraction. For autophagy assessment, the cells were plated in six-well plates at a density of 1x10^6^ cells/well, and allowed to adhere for at least 24 h. Then the growth medium was replaced with fresh growth medium for the control cells or with 20 nM bafilomycin A1 (BaF – autophagy inducer) with and without 2mg/ml of Beva, during 48 hours. Western blot was performed as described previously [38, 51]. Incubation with primary antibodies was performed overnight at 4°C. The bound antibodies were visualized by chemiluminescence (Supersignal West Femto kit; Pierce).

### Extracellular lactate and glucose measurements

Cells were plated in 48 well plates at a density of 3x10^4^ cells per well and allowed to adhere overnight. Lactate and glucose contents were analyzed in cell culture medium after 48h of 2mg/ml Beva treatment, using commercial kits (Spinreact), as described [38, 39]. For these time points, the total protein (expressed as total biomass) was assessed using the sulforhodamine B assay (SRB, TOX-6, Sigma-Aldrich). Results are expressed as total μg/ total biomass.

### ROS production and mitochondrial activity

Cells were plated in 6 well plate at a density of 2.5x10^5^ cell/well and treated with 2mg/ml Beva during 48 hours. Then, cells were incubated with the molecular probes during 2h at 37°, in dark. After, molecular probe incubation, the cells were washed with PBS and collected to flow cytometry analysis (LSRII, BD Biosciences). For measurement of ROS content, cells were incubated with 200nM of Dihydroethidium (DHE, Molecular Probes). Mitochondrial polarization was assessed by 200nM Mitotracker Red and mitochondrial mass by 200nM Mitotracker Green (Molecular probes). The mitochondrial activity was represented as the ratio mitochondria polarization: mitochondria mass.

### Glycogen accumulation

Glycogen accumulation in cells was observed through the Periodic and Schiff (PAS) staining. The cells were plated at density of 5x10^4^ cell/well and allowed to adhere overnight. Then 2mg/ml Beva treatment was performed in DMEM 0.5% FBS during 48hours. After that cells were fixed in 4% paraformaldehyde (PFA) for 15minutes, followed the incubation with periodic acid for 5minutes and then Schiff reagent for 10 minutes. Periodic acid oxidizes the vicinal diols in glycogen and form a pair of aldehydes with reacts with Schiff reagent and give a purple-magenta color. In the end, hematoxylin was used to do counterstaining.

### Chicken chorioallantoic membrane (CAM) assay

CAM assay was performed as described previously [52]. At 9 day of development, 2x10^6^ cells were injected with Matrigel on CAM, allowing the tumor formation during 4 days. On 13 day, tumors were photographed and treated with the respective conditions: control group, 2mg/ml Beva group, 1mM 2-Deoxy-D-glucose (2DG) group or 1mM 2-DG + 2mg/ml Beva. After 96 hours, CAMs were dissected, fixed in 4% paraformaldehyde and photographed to vessel counting and perimeter measurement. Vessels was counted using the ImageJ 14.1 version software. The results of the perimeter at the 17 day were expressed in fold change in relation to the mean perimeter of the tumors at the day 13 (considered as 1). The results of the number of blood vessels at day 17 were expressed in fold change in relation to the control (considered as 1).

### Statistical analysis

For the *in vitro* and *in vivo* studies, the GraphPad prism 5 software was used to statistical analysis, with the Student’s *t* test or the One-Way ANOVA test, considering significant values *p*<0.05.

## SUPPLEMENTARY MATERIALS FIGURES



## References

[1] Agnihotri S, Burrell KE, Wolf A, Jalali S, Hawkins C, Rutka JT, Zadeh G (2013). Glioblastoma, a brief review of history, molecular genetics, animal models and novel therapeutic strategies. Arch Immunol Ther Exp (Warsz).

[2] Ostrom QT, Bauchet L, Davis FG, Deltour I, Fisher JL, Langer CE, Pekmezci M, Schwartzbaum JA, Turner MC, Walsh KM, Wrensch MR, Barnholtz-Sloan JS (2014). The epidemiology of glioma in adults: a “state of the science” review. Neuro-oncol.

[3] Weller M, Pfister SM, Wick W, Hegi ME, Reifenberger G, Stupp R (2013). Molecular neuro-oncology in clinical practice: a new horizon. Lancet Oncol.

[4] Tanaka S, Louis DN, Curry WT, Batchelor TT, Dietrich J (2013). Diagnostic and therapeutic avenues for glioblastoma: no longer a dead end?. Nat Rev Clin Oncol.

[5] Yang LJ, Zhou CF, Lin ZX (2014). Temozolomide and radiotherapy for newly diagnosed glioblastoma multiforme: a systematic review. Cancer Invest.

[6] Stupp R, Mason WP, van den Bent MJ, Weller M, Fisher B, Taphoorn MJ, Belanger K, Brandes AA, Marosi C, Bogdahn U, Curschmann J, Janzer RC, Ludwin SK, European Organisation for Research and Treatment of Cancer Brain Tumor and Radiotherapy Groups, and National Cancer Institute of Canada Clinical Trials Group (2005). Radiotherapy plus concomitant and adjuvant temozolomide for glioblastoma. N Engl J Med.

[7] Weller M, van den Bent M, Hopkins K, Tonn JC, Stupp R, Falini A, Cohen-Jonathan-Moyal E, Frappaz D, Henriksson R, Balana C, Chinot O, Ram Z, Reifenberger G, European Association for Neuro-Oncology (EANO) Task Force on Malignant Glioma (2014). EANO guideline for the diagnosis and treatment of anaplastic gliomas and glioblastoma. Lancet Oncol.

[8] Stupp R, Hegi ME, Mason WP, van den Bent MJ, Taphoorn MJ, Janzer RC, Ludwin SK, Allgeier A, Fisher B, Belanger K, Hau P, Brandes AA, Gijtenbeek J, European Organisation for Research and Treatment of Cancer Brain Tumour and Radiation Oncology Groups, and National Cancer Institute of Canada Clinical Trials Group (2009). Effects of radiotherapy with concomitant and adjuvant temozolomide versus radiotherapy alone on survival in glioblastoma in a randomised phase III study: 5-year analysis of the EORTC-NCIC trial. Lancet Oncol.

[9] Jürgensmeier JM, Eder JP, Herbst RS (2014). New strategies in personalized medicine for solid tumors: molecular markers and clinical trial designs. Clin Cancer Res.

[10] Jain RK, di Tomaso E, Duda DG, Loeffler JS, Sorensen AG, Batchelor TT (2007). Angiogenesis in brain tumours. Nat Rev Neurosci.

[11] Soda Y, Myskiw C, Rommel A, Verma IM (2013). Mechanisms of neovascularization and resistance to anti-angiogenic therapies in glioblastoma multiforme. J Mol Med (Berl).

[12] Hanahan D, Folkman J (1996). Patterns and emerging mechanisms of the angiogenic switch during tumorigenesis. Cell.

[13] Dvorak HF (2002). Vascular permeability factor/vascular endothelial growth factor: a critical cytokine in tumor angiogenesis and a potential target for diagnosis and therapy. J Clin Oncol.

[14] Krämer I, Lipp HP (2007). Bevacizumab, a humanized anti-angiogenic monoclonal antibody for the treatment of colorectal cancer. J Clin Pharm Ther.

[15] Gentzler RD, Yentz SE, Patel JD (2013). Bevacizumab in advanced NSCLC: chemotherapy partners and duration of use. Curr Treat Options Oncol.

[16] Gil-Gil MJ, Mesia C, Rey M, Bruna J (2013). Bevacizumab for the treatment of glioblastoma. Clin Med Insights Oncol.

[17] Lai A, Tran A, Nghiemphu PL, Pope WB, Solis OE, Selch M, Filka E, Yong WH, Mischel PS, Liau LM, Phuphanich S, Black K, Peak S (2011). Phase II study of bevacizumab plus temozolomide during and after radiation therapy for patients with newly diagnosed glioblastoma multiforme. J Clin Oncol.

[18] Friedman HS, Prados MD, Wen PY, Mikkelsen T, Schiff D, Abrey LE, Yung WK, Paleologos N, Nicholas MK, Jensen R, Vredenburgh J, Huang J, Zheng M, Cloughesy T (2009). Bevacizumab alone and in combination with irinotecan in recurrent glioblastoma. J Clin Oncol.

[19] Gil MJ, de Las Peñas R, Reynés G, Balañá C, Peréz-Segura P, García-Velasco A, Mesia C, Gallego O, Fernández-Chacón C, Martínez-García M, Herrero A, Andrés R, Benavides M (2012). Bevacizumab plus irinotecan in recurrent malignant glioma shows high overall survival in a multicenter retrospective pooled series of the Spanish Neuro-Oncology Research Group (GEINO). Anticancer Drugs.

[20] Hasselbalch B, Lassen U, Hansen S, Holmberg M, Sørensen M, Kosteljanetz M, Broholm H, Stockhausen MT, Poulsen HS (2010). Cetuximab, bevacizumab, and irinotecan for patients with primary glioblastoma and progression after radiation therapy and temozolomide: a phase II trial. Neuro Oncol.

[21] Reardon DA, Desjardins A, Peters K, Gururangan S, Sampson J, Rich JN, McLendon R, Herndon JE, Marcello J, Threatt S, Friedman AH, Vredenburgh JJ, Friedman HS (2011). Phase II study of metronomic chemotherapy with bevacizumab for recurrent glioblastoma after progression on bevacizumab therapy. J Neurooncol.

[22] Sathornsumetee S, Desjardins A, Vredenburgh JJ, McLendon RE, Marcello J, Herndon JE, Mathe A, Hamilton M, Rich JN, Norfleet JA, Gururangan S, Friedman HS, Reardon DA (2010). Phase II trial of bevacizumab and erlotinib in patients with recurrent malignant glioma. Neuro-oncol.

[23] Sathornsumetee S, Cao Y, Marcello JE, Herndon JE, McLendon RE, Desjardins A, Friedman HS, Dewhirst MW, Vredenburgh JJ, Rich JN (2008). Tumor angiogenic and hypoxic profiles predict radiographic response and survival in malignant astrocytoma patients treated with bevacizumab and irinotecan. J Clin Oncol.

[24] Keunen O, Johansson M, Oudin A, Sanzey M, Rahim SA, Fack F, Thorsen F, Taxt T, Bartos M, Jirik R, Miletic H, Wang J, Stieber D (2011). Anti-VEGF treatment reduces blood supply and increases tumor cell invasion in glioblastoma. Proc Natl Acad Sci USA.

[25] Granja S, Pinheiro C, Reis RM, Martinho O, Baltazar F (2015). Glucose addiction in cancer therapy: advances and drawbacks. Curr Drug Metab.

[26] Xu J, Wang J, Xu B, Ge H, Zhou X, Fang JY (2013). Colorectal cancer cells refractory to anti-VEGF treatment are vulnerable to glycolytic blockade due to persistent impairment of mitochondria. Mol Cancer Ther.

[27] Bergers G, Hanahan D (2008). Modes of resistance to anti-angiogenic therapy. Nat Rev Cancer.

[28] De Bock K, Mazzone M, Carmeliet P (2011). Antiangiogenic therapy, hypoxia, and metastasis: risky liaisons, or not?. Nat Rev Clin Oncol.

[29] Hattingen E, Jurcoane A, Bähr O, Rieger J, Magerkurth J, Anti S, Steinbach JP, Pilatus U (2011). Bevacizumab impairs oxidative energy metabolism and shows antitumoral effects in recurrent glioblastomas: a 31P/1H MRSI and quantitative magnetic resonance imaging study. Neuro-oncol.

[30] McIntyre A, Harris AL (2015). Metabolic and hypoxic adaptation to anti-angiogenic therapy: a target for induced essentiality. EMBO Mol Med.

[31] McIntyre A, Patiar S, Wigfield S, Li JL, Ledaki I, Turley H, Leek R, Snell C, Gatter K, Sly WS, Vaughan-Jones RD, Swietach P, Harris AL (2012). Carbonic anhydrase IX promotes tumor growth and necrosis *in vivo* and inhibition enhances anti-VEGF therapy. Clin Cancer Res.

[32] Yopp AC, Schwartz LH, Kemeny N, Gultekin DH, Gönen M, Bamboat Z, Shia J, Haviland D, D’Angelica MI, Fong Y, DeMatteo RP, Allen PJ, Jarnagin WR (2011). Antiangiogenic therapy for primary liver cancer: correlation of changes in dynamic contrast-enhanced magnetic resonance imaging with tissue hypoxia markers and clinical response. Ann Surg Oncol.

[33] DeLay M, Jahangiri A, Carbonell WS, Hu YL, Tsao S, Tom MW, Paquette J, Tokuyasu TA, Aghi MK (2012). Microarray analysis verifies two distinct phenotypes of glioblastomas resistant to antiangiogenic therapy. Clin Cancer Res.

[34] Mehta S, Hughes NP, Buffa FM, Li SP, Adams RF, Adwani A, Taylor NJ, Levitt NC, Padhani AR, Makris A, Harris AL (2011). Assessing early therapeutic response to bevacizumab in primary breast cancer using magnetic resonance imaging and gene expression profiles. J Natl Cancer Inst Monogr.

[35] Marchiq I, Pouysségur J (2016). Hypoxia, cancer metabolism and the therapeutic benefit of targeting lactate/H(+) symporters. J Mol Med (Berl).

[36] Curtarello M, Zulato E, Nardo G, Valtorta S, Guzzo G, Rossi E, Esposito G, Msaki A, Pastò A, Rasola A, Persano L, Ciccarese F, Bertorelle R (2015). VEGF-targeted therapy stably modulates the glycolytic phenotype of tumor cells. Cancer Res.

[37] Fack F, Espedal H, Keunen O, Golebiewska A, Obad N, Harter PN, Mittelbronn M, Bähr O, Weyerbrock A, Stuhr L, Miletic H, Sakariassen PØ, Stieber D (2015). Bevacizumab treatment induces metabolic adaptation toward anaerobic metabolism in glioblastomas. Acta Neuropathol.

[38] Miranda-Gonçalves V, Granja S, Martinho O, Honavar M, Pojo M, Costa BM, Pires MM, Pinheiro C, Cordeiro M, Bebiano G, Costa P, Reis RM, Baltazar F (2016). Hypoxia-mediated upregulation of MCT1 expression supports the glycolytic phenotype of glioblastomas. Oncotarget.

[39] Miranda-Gonçalves V, Honavar M, Pinheiro C, Martinho O, Pires MM, Pinheiro C, Cordeiro M, Bebiano G, Costa P, Palmeirim I, Reis RM, Baltazar F (2013). Monocarboxylate transporters (MCTs) in gliomas: expression and exploitation as therapeutic targets. Neuro-oncol.

[40] Favaro E, Bensaad K, Chong MG, Tennant DA, Ferguson DJ, Snell C, Steers G, Turley H, Li JL, Günther UL, Buffa FM, McIntyre A, Harris AL (2012). Glucose utilization via glycogen phosphorylase sustains proliferation and prevents premature senescence in cancer cells. Cell Metab.

[41] Hu YL, DeLay M, Jahangiri A, Molinaro AM, Rose SD, Carbonell WS, Aghi MK (2012). Hypoxia-induced autophagy promotes tumor cell survival and adaptation to antiangiogenic treatment in glioblastoma. Cancer Res.

[42] Liang J, Piao Y, Henry V, Tiao N, de Groot JF (2015). Interferon-regulatory factor-1 (IRF1) regulates bevacizumab induced autophagy. Oncotarget.

[43] Hong X, Jiang F, Kalkanis SN, Zhang ZG, Zhang X, Zheng X, Mikkelsen T, Jiang H, Chopp M (2007). Decrease of endogenous vascular endothelial growth factor may not affect glioma cell proliferation and invasion. J Exp Ther Oncol.

[44] Perera RM, Zoncu R, Johns TG, Pypaert M, Lee FT, Mellman I, Old LJ, Toomre DK, Scott AM (2007). Internalization, intracellular trafficking, and biodistribution of monoclonal antibody 806: a novel anti-epidermal growth factor receptor antibody. Neoplasia.

[45] Guan H, Jia SF, Zhou Z, Stewart J, Kleinerman ES (2005). Herceptin down-regulates HER-2/neu and vascular endothelial growth factor expression and enhances taxol-induced cytotoxicity of human Ewing’s sarcoma cells *in vitro* and *in vivo*. Clin Cancer Res.

[46] Hasan MR, Ho SH, Owen DA, Tai IT (2011). Inhibition of VEGF induces cellular senescence in colorectal cancer cells. Int J Cancer.

[47] Hartwich J, Orr WS, Ng CY, Spence Y, Morton C, Davidoff AM (2013). HIF-1α activation mediates resistance to anti-angiogenic therapy in neuroblastoma xenografts. J Pediatr Surg.

[48] Rapisarda A, Hollingshead M, Uranchimeg B, Bonomi CA, Borgel SD, Carter JP, Gehrs B, Raffeld M, Kinders RJ, Parchment R, Anver MR, Shoemaker RH, Melillo G (2009). Increased antitumor activity of bevacizumab in combination with hypoxia inducible factor-1 inhibition. Mol Cancer Ther.

[49] Kumar K, Wigfield S, Gee HE, Devlin CM, Singleton D, Li JL, Buffa F, Huffman M, Sinn AL, Silver J, Turley H, Leek R, Harris AL, Ivan M (2013). Dichloroacetate reverses the hypoxic adaptation to bevacizumab and enhances its antitumor effects in mouse xenografts. J Mol Med (Berl).

[50] Silva-Oliveira RJ, Silva VA, Martinho O, Cruvinel-Carloni A, Melendez ME, Rosa MN, de Paula FE, de Souza Viana L, Carvalho AL, Reis RM (2016). Cytotoxicity of allitinib, an irreversible anti-EGFR agent, in a large panel of human cancer-derived cell lines: KRAS mutation status as a predictive biomarker. Cell Oncol (Dordr).

[51] Martinho O, Silva-Oliveira R, Miranda-Gonçalves V, Clara C, Almeida JR, Carvalho AL, Barata JT, Reis RM (2013). *In Vitro* and *In Vivo* Analysis of RTK Inhibitor Efficacy and Identification of Its Novel Targets in Glioblastomas. Transl Oncol.

[52] Martinho O, Silva-Oliveira R, Cury FP, Barbosa AM, Granja S, Evangelista AF, Marques F, Miranda-Gonçalves V, Cardoso-Carneiro D, de Paula FE, Zanon M, Scapulatempo-Neto C, Moreira MA (2017). HER Family Receptors are Important Theranostic Biomarkers for Cervical Cancer: Blocking Glucose Metabolism Enhances the Therapeutic Effect of HER Inhibitors. Theranostics.

