# Supplementary material for: Metabolic alterations underlying Bevacizumab therapy in glioblastoma cells
Source: Oncotarget. 2017 Oct 10;8(61):103657–70. doi: 10.18632/oncotarget.21761 (PMC5732757; doi:10.18632/oncotarget.21761)
Supplement: Supplementary file 1 [file oncotarget-08-103657-s001.pdf]

Metabolic alterations underlying Bevacizumab therapy in glioblastoma cells

SUPPLEMENTARY MATERIALS

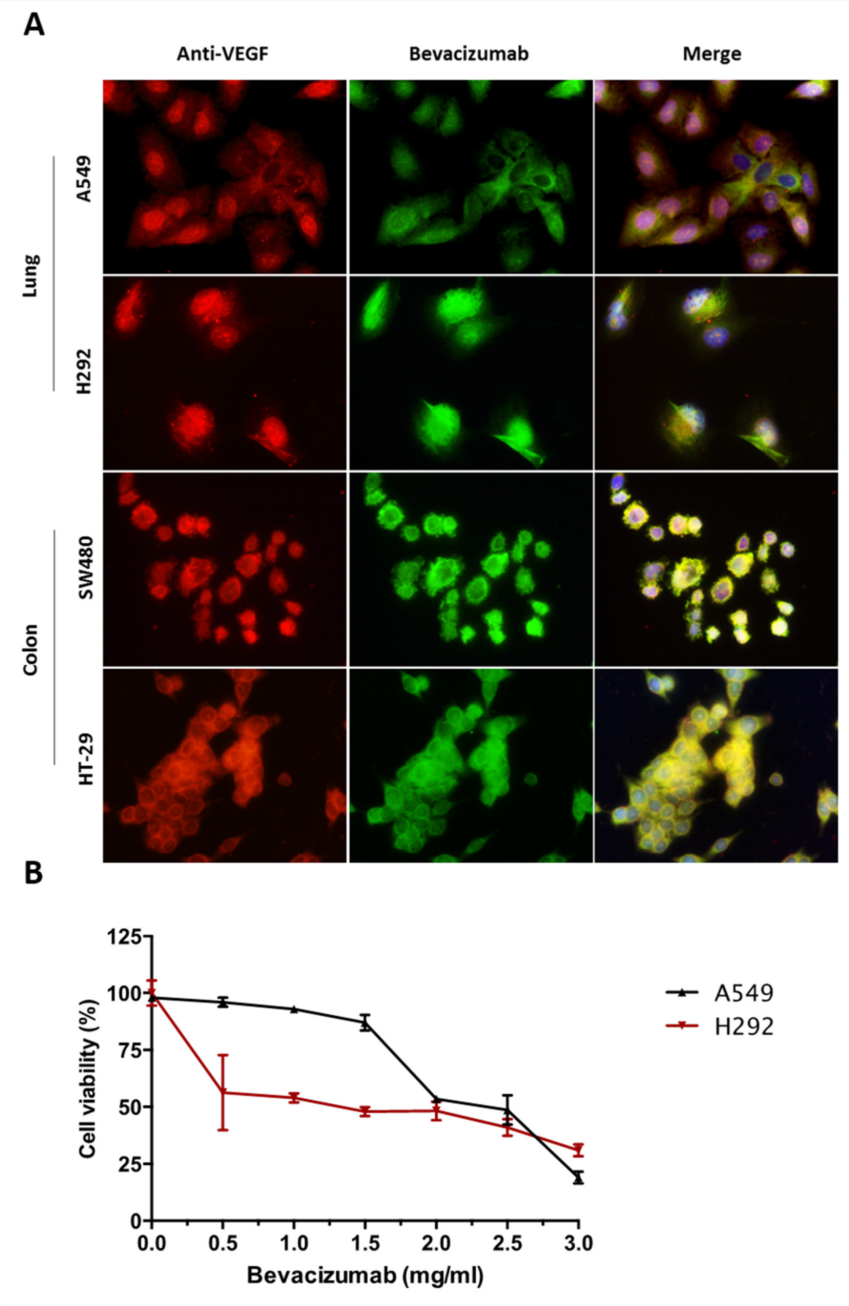

Supplementary Figure 1: *In vitro* effect of Bevacizumab in colon and lung cancer cell lines. (A) Immunofluorescence for VEGFA using a specific anti-VEGF antibody and Beva as primary antibodies. Pictures were taken at 400x in an Olympus fluorescence microscope. **(B)** Cell viability of lung cancer cell lines exposed to increasing concentrations of Beva was assessed by MTS assay at 72 hours of treatment; results are from three independent assays, each one in triplicates.

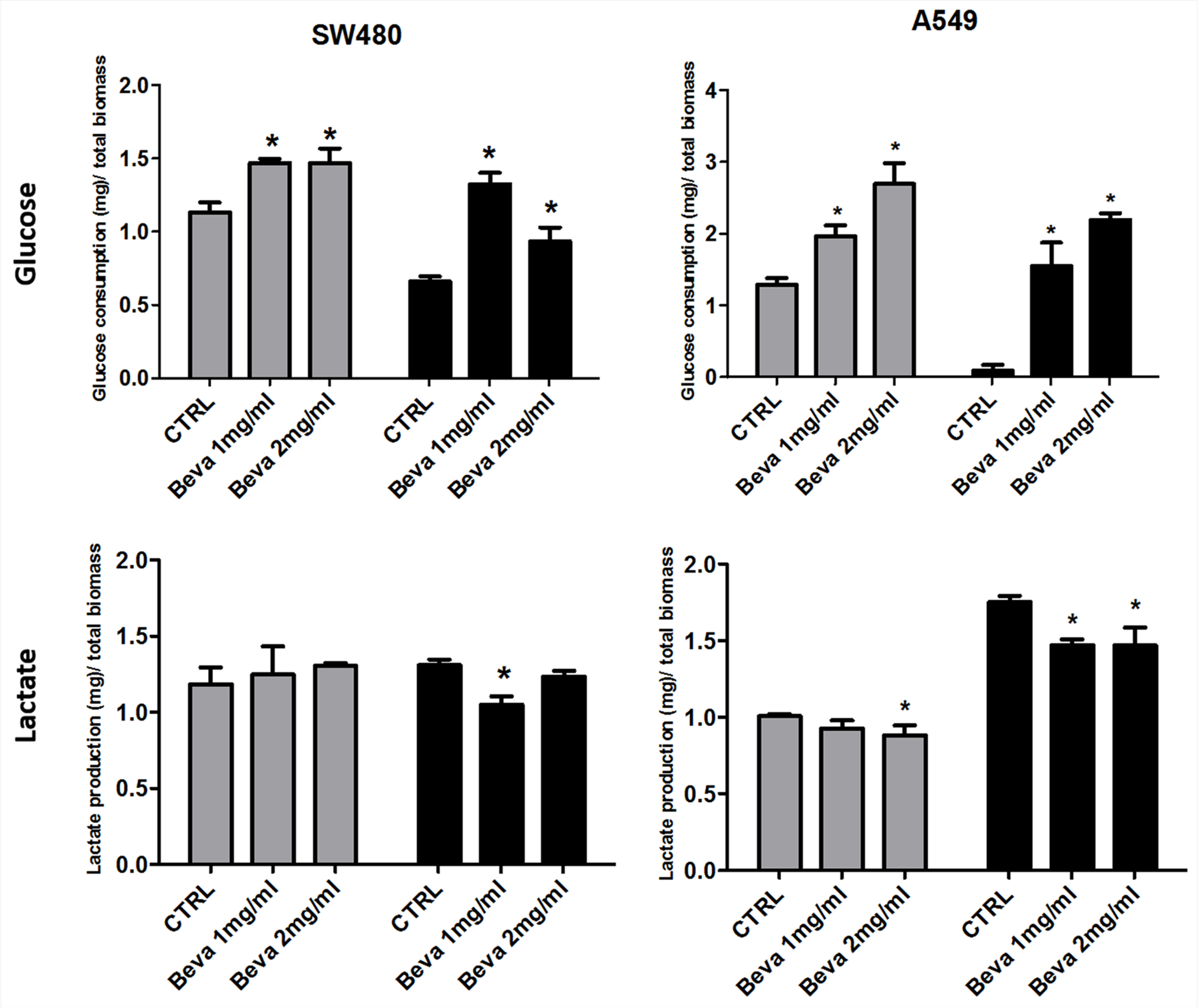

Supplementary Figure 2: Effect of Bevacizumab treatment on glucose metabolism of colon and lung cancer cells. Rates of glucose consumption and lactate production on SW480 (colon) and A549 (lung) cell lines after 48 hours of treatment. The rates of glucose consumption were increased in both cell lines without increasing the levels of exported lactate. Results are representative of three independent experiments, each one in triplicates; $^{*}p<0.05$, $^{****}p\leq0.001$.
